# Socioeconomic Determinants of Antibiotic Consumption in the State of São Paulo, Brazil: The Effect of Restricting Over-The-Counter Sales

**DOI:** 10.1371/journal.pone.0167885

**Published:** 2016-12-12

**Authors:** Breno S. Kliemann, Anna S. Levin, M. Luísa Moura, Icaro Boszczowski, James J. Lewis

**Affiliations:** 1 Department of Population Health, Faculty of Epidemiology and Population Health, London School of Hygiene & Tropical Medicine, London, United Kingdom; 2 Course of Medicine, Health Sciences Sector, Federal University of Paraná, Curitiba, Brazil; 3 Infection Control Department, Hospital das Clínicas, University of São Paulo, São Paulo, Brazil; 4 Department of Infectious Diseases and LIM54, Faculdade de Medicina, University of São Paulo, São Paulo, Brazil; 5 MRC Tropical Epidemiology Group, Department of Infectious Disease Epidemiology, Faculty of Epidemiology and Population Health, London School of Hygiene & Tropical Medicine, London, United Kingdom; Western General Hospital, UNITED KINGDOM

## Abstract

**Background:**

Improper antibiotic use is one of the main drivers of bacterial resistance to antibiotics, increasing infectious diseases morbidity and mortality and raising costs of healthcare. The level of antibiotic consumption has been shown to vary according to socioeconomic determinants (SED) such as income and access to education. In many Latin American countries, antibiotics could be easily purchased without a medical prescription in private pharmacies before enforcement of restrictions on over-the-counter (OTC) sales in recent years. Brazil issued a law abolishing OTC sales in October 2010. This study seeks to find SED of antibiotic consumption in the Brazilian state of São Paulo (SSP) and to estimate the impact of the 2010 law.

**Methods:**

Data on all oral antibiotic sales having occurred in the private sector in SSP from 2008 to 2012 were pooled into the 645 municipalities of SSP. Linear regression was performed to estimate consumption levels that would have occurred in 2011 and 2012 if no law regulating OTC sales had been issued in 2010. These values were compared to actual observed levels, estimating the effect of this law. Linear regression was performed to find association of antibiotic consumption levels and of a greater effect of the law with municipality level data on SED obtained from a nationwide census.

**Results:**

Oral antibiotic consumption in SSP rose from 8.44 defined daily doses per 1,000 inhabitants per day (DID) in 2008 to 9.95 in 2010, and fell to 8.06 DID in 2012. Determinants of a higher consumption were higher human development index, percentage of urban population, density of private health establishments, life expectancy and percentage of females; lower illiteracy levels and lower percentage of population between 5 and 15 years old. A higher percentage of females was associated with a stronger effect of the law.

**Conclusions:**

SSP had similar antibiotic consumption levels as the whole country of Brazil, and they were effectively reduced by the policy.

## Introduction

Although infectious diseases continue to be a major cause of death worldwide [1], the development of new antibiotics is in decline, and no new therapeutic classes have been discovered since the 1970s. [2] The occurrence of antimicrobial resistance (AMR) in the community setting is becoming increasingly common. [3] Overuse of antibiotics in clinical settings is currently recognised as one of the main drivers of emerging antibiotic resistance worldwide. [4] Misuse of antibiotics, by erroneous prescribing or noncompliance may further contribute to antibiotic resistance. [5] Goossens *et al*. (2005) [6] analysed levels of outpatient antibiotic use in 26 European countries, finding an ecological association between antibiotic consumption and rates of AMR—both were higher in France and Southern European countries and lower in the Netherlands and Northern European countries.

Different rates of antibiotic use across regions may occur due to differences in the incidence of infectious diseases, in the availability of antibiotics, in patient-related factors, in antibiotic prescribing or dispensing. [5] These factors are often inter-related and may be affected by several distal determinants. For instance, cultural aspects and the pressure of the pharmaceutical companies may act through raising a patient’s demand for antibiotics, and the patient’s demand for antibiotics has been shown to influence the doctor’s decision on whether to prescribe it or not. [4,5] Patient-related factors are often related to knowledge about antibiotics’ risks and benefits, relationship with physicians, and economic factors. [5] Additionally, differences in the incidence of infectious disease are the main reason for seasonal variations in antibiotic consumption [7]; and, in some low-income countries, areas may have lower antibiotic consumption rates because of lack of availability of antibiotics. [8] Many socioeconomic determinants (SED), such as income and education levels, have been shown to be possible predictors of antibiotic consumption. [9–11]

In most Latin American countries (LAC), antibiotics are dispensed only with a medical prescription in the public sector. In private pharmacies, however, it was possible to buy them without prescriptions—i.e., over-the-counter (OTC). While in some settings OTC sales may be essential to allow the access to medicines for people who do not have access to physicians, the possibility of self-medication increases the risk of overuse and misuse of antibiotics and hence of resistance. [12] Some LAC have, in recent years, promoted policies enforcing their regulation on antibiotic sales and prohibiting OTC sales. Chile abolished OTC sales in 1999; and Colombia did the same in 2005, but only in the capital district of Bogotá. Venezuela prohibited OTC sales nationwide for some classes of antibiotics (macrolides, quinolones and third-generation cephalosporins) in 2006; and Mexico prohibited all OTC sales in 2010. [13–18]

Brazil also issued a law abolishing OTC sales in October 2010. [19] It enforced the requirement of medical prescriptions for antibiotic sales in private pharmacies, demanding a copy of the prescription to be retained in the pharmacy for every sale. Before this intervention, 46% of antibiotic sales in Brazil were reported to occur without a medical prescription. [12] Wirtz *et al*. (2010) [20], in an analysis of retail sales between 1997 and 2007, reported that Brazil had the lowest antibiotic consumption rate among eight LAC, increasing from 6.51 to 7.01 defined daily doses per 1,000 inhabitants per day during this time period. In a review of global antibiotic consumption trends, Van Boeckel *et al*. (2014) [7] noticed that, although being among the countries in the lowest interval (1 to 8 standard units per person) of antibiotic consumption level in 2010, Brazil was in one of the highest intervals of consumption compound annual growth rate (2.5 to 6) between 2000 and 2010.

The Brazilian state of São Paulo (SSP) is Brazil’s most populous state, with 41,262,199 people in 2010, and its 645 municipalities are grouped in 15 meso-regions. [21] There are, however, few studies on variations in antibiotic use within SSP and their SED, with these studies usually focusing on single or few municipalities. [22–26] There are also little data about the levels and the evolution of AMR within SSP. Kiffer *et al*. (2011) [22] showed the emergence of ciprofloxacin-resistant *E*. *coli* clusters to be spatially correlated to areas of higher antibiotic consumption within the city of São Paulo—resistant cluster emergence was related to an antibiotic use density of 5 to 9 defined daily doses per 1,000 inhabitants-day. In the same study, out of 4,372 cases of urinary tract infection caused by *E*. *coli*, identified from two outpatient centres, 723 were resistant to ciprofloxacin. Miranda *et al*. (2014) [26] studied the rate of antibiotic resistance to urinary tract infections in the city of São Paulo during two periods: 2005–2006 and 2010–2011. They found a significant increase in bacterial resistance to most antibiotics between these two periods, particularly to fluoroquinolones (90.1% of infections were susceptible to ciprofloxacin in 2005–2006 vs 83.4% in 2010–2011). Global susceptibility of *E*. *coli*, the most common urinary tract infection agent, decreased from 88.3% to 86.1%, and resistance was greater among men and patients older than 65 years.

The present study is, to our knowledge, the first to focus on SED of antibiotic consumption specifically in SSP and to analyse the micro-level variations in the effect of Brazil’s policy of OTC sales abolishment. We have three objectives:

1. To estimate the impact of the OTC sales prohibition on pharmacy antibiotic consumption in SSP.
2. To identify regions with higher levels of pharmacy antibiotic consumption, both before and after implementation of the policy.
3. To identify determinants of higher levels of pharmacy antibiotic consumption and of the policy effect.

## Materials and Methods

Data on antimicrobial annual wholesale sales across SSP were provided by Intercontinental Medical Statistics Health Brazil, a healthcare consultancy organisation for pharmaceutical industry research. It consisted of sales to establishments such as pharmacies, clinics and hospitals; having occurred from 2008 to 2012. Data were given by defined daily dose (DDD), by municipality and by year. Data on total antibiotic consumption in SSP (not divided by municipality) were also available on a monthly basis. DDD is the suggested unit for measurement of drug consumption in a population, being defined as:

“The assumed average maintenance dose per day for a drug used for its main indication in adults”

(WHO Collaborating Centre for Drug Statistics Methodology, 2014) [27].

As antibiotics were already only dispensed with a medical prescription in public healthcare units and hospitals before the 2010 law, this study focuses on antibiotic sales to private pharmacies only. Drugs administered via any other route than oral, such as parenteral antibiotics, were also excluded. Wholesale sales to pharmacies were assumed to reflect accurately outpatient oral antibiotic consumption for the same year and municipality they occurred.

The antibiotic consumption data were pooled with information on SED for each municipality, coming from the nationwide census performed in 2010 by the Brazilian Institute of Geography and Statistics. [28] Information on some variables was also collected from Brazil’s National Health System/Sistema Único de Saúde (SUS) Information Department [29] and Atlas of Human Development in Brazil [30] databases. Data were collected, at municipality level, on the following variables: population, population density, gross domestic product (GDP) per capita, life expectancy at birth, average number of people in the household, Gini coefficient, percentage of population living in urban areas, number of SUS health establishments, number of private health establishments, percentage of females in the population, percentage of population under 5 years old, percentage between 5 and 15 years old, percentage over 60 years old, percentage with completed higher education (out of those over 25 years old), percentage illiterate, number of hospital admittances due to infectious diseases (by municipality of residence), human development index (HDI), geography (meso-region), and whether the municipality belonged or not to São Paulo city and its metropolitan area. These data refer to the year of 2010 (except GDP per capita, which refer to 2012) and were assumed to have remained constant throughout the whole period of 2008 to 2012.

Antibiotic consumption in DDD per 1,000 inhabitants per day (DID) was calculated for the whole SSP in each month, from January 2008 to December 2012. An interrupted time series analysis was performed to calculate the trends of consumption both before and after enforcement of the law, estimating changes in the slope and level of consumption after it.

Annual antibiotic consumption, in DID, was also calculated for each municipality, for three years before the policy implementation: 2008, 2009 and 2010 (as it happened in October 2010, the whole year of 2010 was defined as “before the policy”); and two years after: 2011 and 2012. A linear trend was fitted using the 2008 to 2010 consumption values to estimate the consumption levels that would have occurred in 2011 and 2012 in the counterfactual scenario of no policy having occurred in 2010. These “predicted” values were compared with the actual observed values for 2011 and 2012 to evaluate the effect of the policy in the whole SSP and in each municipality, as follows:

$$\frac{100\;*\;\left(\text{Observed consumption in }2011\text{ and }2012-\text{predicted consumption in }2011\text{ and }2012\right)}{\text{predicted consumption in }2011\text{ and }2012}$$

This percentage reduction in consumption was used as a proxy variable to measure the policy effect. As we had no data on variations in consumption of other medications (e.g., anti-hypertensives) to use as a control group, an assumption was made that there were no other plausible reasons for a difference in consumption during this timeframe, and that this difference has then occurred due to the policy. Where the effect was stronger, observed consumption was smaller if compared to predicted consumption, and hence the difference was more negative.

To identify possible SED of differences in consumption, linear regression models were run with the natural logarithms (the positive skewness of the antibiotic consumption data required a logarithmic transformation) of mean consumption between 2008 and 2010 as the outcome variable, each of the determinants taken from the census as explanatory variables and municipalities as units of analysis. The mean consumption between 2008 and 2010 was chosen for this analysis instead of consumption during the whole period in order to tell apart SED of the consumption itself from SED of the policy effect. First, univariate linear regression models were estimated for each of the explanatory variables. As this study does not aim to test any specific hypothesis, a comprehensive approach was taken and all variables with a p<0.1 in the univariate analysis were selected to be included in a multivariable regression model. [31] The same procedures were performed to search for association between the policy effect (the percentage difference between observed and predicted values) and the explanatory variables, adding mean consumption between 2008 and 2010 as a possible determinant. HDI was not added into the multivariable regression models because it is an index that consists of characteristics that are already evaluated by other variables (GDP per capita, life expectancy and education). Both SUS and private health establishments were added to the model as a larger value of one does not necessarily imply a smaller value of the other. There was no evidence of collinearity and standard errors did not increase highly when both variables were added into the model. Both variables related to education (percentage illiterate and percentage with higher education) were included due to the same reasons. Standardised regression coefficients (subtracting the mean of each variable from its coefficient and then dividing it by the standard deviation) were calculated to give a better idea of the impact each SED had on the outcomes.

An analysis of variance (ANOVA) was performed to calculate how both antibiotic consumption and the policy effect have varied according to the 15 meso-regions. All analyses were made using the statistical software STATA 13.0.

Ethical approval was granted for this study by the London School of Hygiene & Tropical Medicine MSc Research Ethics Committee (#9586), and The Faculty of Medicine of the University of São Paulo Research Ethics Committee has given ethical approval for the use of the antibiotic consumption data (#443/11). As the data on pharmacy antimicrobial sales have no individual identification of the consumers, retrospective consent from the participants was not necessary. Exposure data were unlinked with antimicrobial sales data and publicly available at municipality level.

## Results

**Fig 1:**
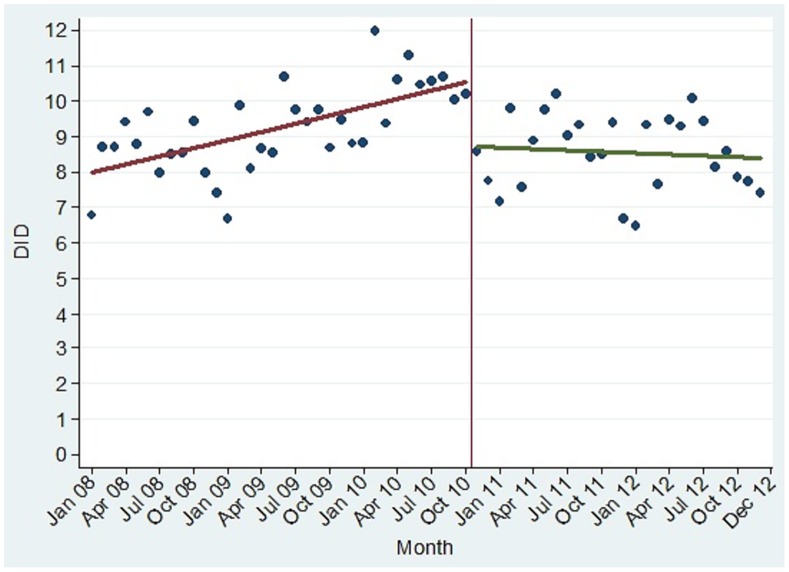
Monthly oral antibiotic consumption in the state of São Paulo. *DID: defined daily doses per 1,000 inhabitants per day. The vertical line represents the issuing of the law.

The monthly consumption of antibiotics in the whole SSP for the five years analysed is depicted in Fig 1, along with the fitted linear trends for before and after the policy. There was a significant change in level of consumption of -1.616 DID (p = 0.002). Consumption before the policy had a trend to increase, with a slope of +0.079 DID per month (p<0.001). After the policy, the trend was to remain stable, with a slope of -0.025 DID per month (p = 0.3553). This change in the slope of consumption, of -0.103 DID per month, was also significant (p = 0.001). Annual antibiotic consumption rose from 8.44 DID in 2008 to 8.76 DID in 2009; and peaked at 9.95 DID in 2010, the year the law was enforced. It fell to 8.58 DID in 2011 and to 8.06 DID in 2012. By extrapolating the trend from 2008–2010, the estimated consumption would have been 10.56 DID in 2011 and 11.31 DID in 2012 in the absence of the law. Hence, the policy effect was a reduction in consumption of 23.91%. For the great majority of municipalities (86%), observed consumption was lower than predicted without the law.

**Fig 2:**
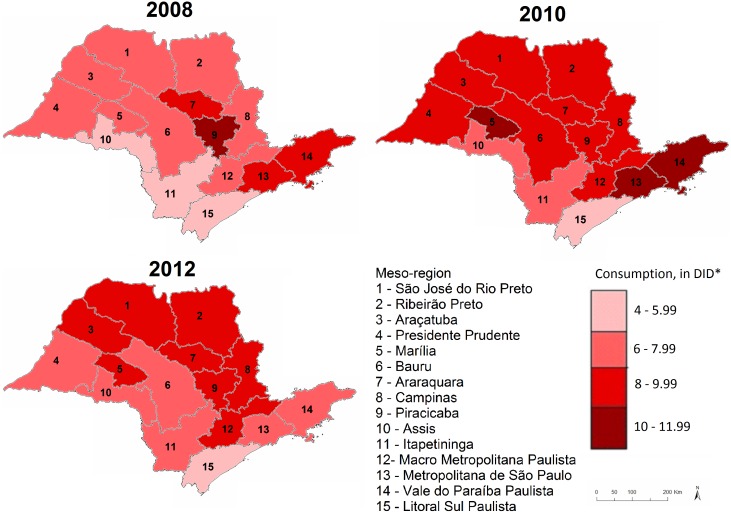
Oral antibiotic consumption by meso-region in 2008, 2010 and 2012. *DID: defined daily doses per 1,000 inhabitants per day.

Furthermore, one-way ANOVA tests provided strong statistical evidence (p<0.001) supporting the presence of significant variations in oral antibiotic consumption across the 15 different meso-regions. Fig 2 shows how consumption varied across the meso-regions of SSP in three different years. Although most meso-regions had a consumption level ranging from 4 to 8 DID in 2008, only three remained with this level two years later—Assis (7.08 DID), Itapetininga (7.67 DID) and Litoral Sul Paulista (5.28 DID). In three other meso-regions consumption levels were higher than 10 DID in 2010: Marília (11.20 DID), Metropolitana de São Paulo (10.35 DID) and Vale do Paraíba Paulista (10.19 DID). By 2012, consumption had decreased in all meso-regions, peaking at 9 DID in Campinas. Litoral Sul Paulista showed the lowest consumption level throughout the entire period, changing from 4.24 DID in 2008 to 4.17 DID in 2012. The consumptions levels for each meso-region during the five years analysed are shown in Table 1. One-way ANOVA tests also provided very strong evidence (p<0.001) for a variation in the policy effect across meso-regions.

**Table 1:** Annual oral antibiotic consumption in São Paulo and its meso-regions, by DID*.

Meso-region	Year				
	2008	2009	2010	2011	2012
Araçatuba	6.98	7.89	9.54	8.01	8.02
Araraquara	8.11	8.36	9.98	9.03	8.99
Assis	5.14	5.75	7.08	6.62	6.24
Bauru	6.56	7.42	8.79	7.76	7.73
Campinas	7.58	8.23	9.95	9.77	9.00
Itapetininga	5.37	6.28	7.67	7.18	6.37
Litoral Sul Paulista	4.24	4.45	5.28	4.33	4.17
Macro Metropolitana Paulista	7.91	8.60	9.70	8.90	8.35
Marília	7.52	8.98	11.20	8.87	8.64
Metropolitana de São Paulo	9.14	9.39	10.35	8.61	7.87
Piracicaba	11.71	8.78	9.69	9.37	8.76
Presidente Prudente	6.56	7.49	8.92	7.56	7.87
Ribeirão Preto	7.83	8.24	9.68	8.33	8.83
São José do Rio Preto	7.88	8.04	9.91	8.34	8.51
Vale do Paraíba Paulista	8.17	9.04	10.19	8.36	7.96
**Total**	**8.44**	**8.76**	**9.95**	**8.58**	**8.06**

*DID: defined daily doses per 1,000 inhabitants per day.

There was very strong statistical evidence (p<0.001) in favour of the following as predictors of higher antibiotic consumption in SSP (Table 2): HDI, percentage of population with higher education, density of private health establishments, percentage of urban population, lower levels of illiteracy, life expectancy, percentage of females in the population, lower percentage of population between 5 and 15 years old, higher Gini coefficient (i.e., income inequality), lower density of SUS health establishments, lower percentage of population under 5 years old and higher population density. Higher consumption was also statistically associated, with a p<0.05, to a larger population, to a higher GDP per capita, and to whether the municipality belonged or not to São Paulo city metropolitan area (the consumption being higher inside the metropolitan area). Only five variables presented R^2^ values higher than 0.25: HDI, percentage with higher education, density of private health establishments, percentage urban and percentage illiterate.

**Table 2:** Linear regression results for univariate analysis.

Explanatory Variables	Antibiotic consumption				Policy effect			
	exp(b)*	p*	R^2^*	β*	b*	p*	R^2^*	β*
HDI** (%)	1.151	<0.001	0.3445	0.587	-4.483	0.075	0.0050	-0.071
Population with higher education (% out of those aged 25 years or more)	1.113	<0.001	0.3277	0.572	-4.501	0.021	0.0084	-0.091
Private health establishments (per 100,000 people)	1.032	<0.001	0.2926	0.541	-0.943	0.685	0.0039	-0.062
% urban	1.030	<0.001	0.2894	0.538	-1.911	0.001	0.0173	-0.132
% illiterate	0.857	<0.001	0.2806	-0.530	1.050	0.732	0.0002	0.014
Life expectancy	1.315	<0.001	0.2126	0.461	-9.684	0.119	0.0038	-0.619
% female	1.109	<0.001	0.1145	0.338	-24.12	<0.001	0.0881	-0.297
Population between 5 and 15 years old (%)	0.900	<0.001	0.0817	-0.286	-7.232	0.062	0.0055	-0.074
Gini coefficient (%)	1.035	<0.001	0.0608	0.247	-1.732	0.226	0.0023	-0.048
SUS** health establishments (per 100,000 people)	0.994	<0.001	0.0304	-0.174	0.449	0.252	0.0021	0.046
Population under 5 years old (%)	0.886	<0.001	0.0221	-0.149	-18.82	0.027	0.0077	-0.088
Population density (thousand inhabitants/km^2^)	1.100	<0.001	0.0216	0.147	-3.213	0.636	0.0004	-0.019
GDP per capita (in 100,000 BRL)**	1.723	0.001	0.0169	0.130	-26.94	0.538	0.0006	-0.024
Population (in millions)	1.233	0.002	0.0151	0.123	-4.799	0.788	0.0001	-0.011
São Paulo and metropolitan area	1.429	0.003	0.0138	0.117	-19.28	31.86	0.0006	-0.024
Hospital admittances due to infectious diseases (per 1,000 people, by municipality of residence)	1.010	0.131	0.0038	0.061	-2.015	0.238	0.0022	-0.047
Average number of people in the household	0.813	0.235	0.0022	-0.047	-10.01	0.828	0.0001	-0.009
Population over 60 years old (%)	1.008	0.446	0.0009	0.030	-3.749	0.159	0.0031	-0.056
Mean consumption 2008–2010	-	-	-	-	-4.965	0.033	0.0072	-0.085

*exp(b): exponent of regression coefficient; b: regression coefficient; R^2^: squared correlation coefficient; β: standardised regression coefficient.

**HDI: Human Development Index; SUS: Sistema Único de Saúde (Brazil’s national health system); GDP: gross domestic product; BRL: Brazilian reais.

In the analysis of the policy effect (Table 2), there was very strong evidence only for the effect of the percentage of females in the population (p<0.001)–higher percentage of females being associated with a more negative difference and thus a stronger policy effect. There was also statistical evidence (p<0.05) for associations of the policy effect with percentage urban, percentage with higher education, population under 5 years old and mean antibiotic consumption in 2008–2010. An increase in each of these factors weakened the effect. However, the standardised coefficients show these associations to have a small impact, and the low R^2^ values show variations in the outcome to have little correlation with any of these variables.

The following factors remained with statistical evidence of an association to a higher antibiotic consumption in the multivariable model (Table 3): higher percentage of urban population (p<0.001), higher density of private health establishments (p<0.001), smaller population between 5 and 15 years old (p<0.001), lower illiteracy (p = 0.002), higher life expectancy (p = 0.011) and larger proportion of females (p = 0.02). The standardised coefficients show that the variable with the greatest effect was percentage of population living in urban areas (β = 0.240), followed by the density of private health establishments (β = 0.215). The multivariable model for antibiotic consumption explained about half of the variation in the consumption across municipalities (R^2^ = 0.5100). The only variable to remain associated to policy effect with good statistical significance was percentage of females (p<0.001), still acting in direction of an increased effect, with a β = -0.320. However, this model failed to explain much of the variation in the outcome, with a R^2^ = 0.0921.

**Table 3:** Multivariable regression models.

**Total antibiotic consumption**	exp(b)*	95% CI*	**P**	**R^2^** *	β*
% urban	1.013	1.009 to 1.018	<0.001	0.5100	0.240
Private health establishments (per 100,000 people)	1.012	1.008 to 1.017	<0.001		0.215
Population between 5 and 15 years old (%)	0.927	0.889 to 0.967	<0.001		-0.205
% illiterate	0.963	0.941 to 0.986	0.002		-0.128
Population with higher education (% out of those aged 25 years or more)	1.020	1.000 to 1.040	0.053		0.104
Life expectancy	1.056	1.012 to 1.101	0.011		0.091
% female	1.027	1.004 to 1.051	0.020		0.088
Gini coefficient (%)	1.007	0.997 to 1.017	0.199		0.049
Population density (thousand inhabitants/km^2^)	0.971	0.926 to 1.019	0.231		-0.045
Population under 5 years old (%)	1.036	0.951 to 1.128	0.415		0.044
SUS health establishments** (per 100,000 people)	0.998	0.996 to 1.001	0.182		-0.042
GDP per capita (in 100,000 BRL)**	0.847	0.663 to 1.084	0.187		-0.040
São Paulo and metropolitan area	1.103	0.880 to 1.383	0.396		0.032
Population (in millions)	1.035	0.934 to 1.148	0.509		0.020
_cons	0.012	0.0005 to 0.3072	0.008		
**Policy effect**	b*	95% CI*	**p**	**R**^**2**^ *	β*
% female	-26.00	-33.88 to -18.11	<0.001	0.0921	-0.320
Population under 5 years old (%)	-19.65	-49.42 to 10.12	0.195		-0.092
Population between 5 and 15 years old (%)	7.699	-6.410 to 21.808	0.284		0.079
Population with higher education(% out of those aged 25 years or more)	2.119	-3.290 to 7.528	0.442		0.043
% urban	0.244	-1.169 to 1.658	0.735		0.017
Mean consumption 2008–2010	-0.764	-6.493 to 4.966	0.794		-0.013
_cons	1,251.72	932.25 to 1,571.2	<0.001		

*exp(b): exponent of regression coefficient; CI: confidence interval; R^2^: squared correlation coefficient; β: standardised regression coefficient.

**SUS: Sistema Único de Saude (Brazil’s national health system); GDP: gross domestic product; BRL: Brazilian reais.

## Discussion

The 2010 law restricting OTC sales was effective in reversing the trend of increasing oral antibiotic consumption in SSP. There was a significant immediate reduction in the level of consumption, and a significant change in slope, causing the trend to remain stable. Santa-Ana-Tellez *et al*. (2013) [15] analysed the impact of this same policy countrywide in Brazil, controlling for the use of anti-hypertensives, and found a significant reduction of 1.35 DID in the level of consumption immediately after the law enforcement—however, no significant change in slope was seen. In the same way, Moura *et al*. (2015) [32] analysed antibiotic consumption in Brazil from 2008 to 2012 and reported a decrease in level of private consumption of 1.87 DID after the policy, with a significant change of -0.05 in the slope, although the trend to increase remained.

Considering annual figures, consumption decreased from 9.95 DID in 2010 to 8.06 in 2012—values consistent with levels for the whole country of Brazil in previous studies: Moura *et al*. (2015) found Brazil consumption level of oral antibiotics to be 8.41 DID in 2010 and 7.52 DID in 2012. [32] According to Wirtz *et al*. (2010) [20], pharmacy antibiotic consumption was 7.01 DID in 2007. Santa-Ana-Tellez *et al*. (2013) [15] noticed an increase in the private sector antibiotic use from 5.7 in 2007 to 8.5 DID in 2012. It is noticeable that, from 2010 to 2012, consumption has decreased more considerably in those meso-regions of SSP which had a larger consumption in 2010 –no meso-regions had consumption levels greater than 10 DID in 2012, and the distribution of antibiotic sales was more even in 2012 than in 2008.

This decline of 1.89 DID in SSP antibiotic consumption is still smaller than the reduction estimated in Chile during the same timeframe after prohibition of OTC sales, from 12.3 DID in 1999 to 8.5 DID in 2000 –there has been, however, a slow but steady rise in Chilean consumption since 2002. [13,33] Wirtz *et al*. (2013) [14] compared trends in private sales data between 1995 and 2009 in Chile and three other LAC: Colombia (where prohibition of OTC sales took place in 2005), Venezuela (prohibited in 2006) and Mexico (which had not prohibited OTC sales at the time of their study). Colombia has seen a reduction from 9.16 DID in 1997 to 6.76 DID in 2009. Although there had not yet been an intervention in Mexico at the time of their data collection, Mexican consumption decreased from 11.6 in 1997 to 9.2 DID in 2009. Venezuela was the only country where the policy did not show effect: antibiotic use increased from 9.99 DID in 1997 to 15.38 DID in 2009.

Pharmacy antibiotic consumption was positively associated with education in this study—municipalities with lower illiteracy levels and higher percentages of population with higher education had higher levels of antibiotic consumption, though only illiteracy remained with strong statistical significance in the multivariable model. This positive association disagrees with previous studies of antibiotic consumption in other countries. Filippini *et al*. (2006) [9] found that cantons with lower educational levels used more antibiotics in Switzerland, and Marra *et al*. (2010) [10] found greater levels of antibiotic consumption in areas with lower education within the Canadian province of British Columbia. It is possible that education acts differently in developing countries such as Brazil—where it may improve the access to medical treatment—than in developed countries such as Switzerland and Canada—where besides education increasing health knowledge and correct antibiotic use, access to medical treatment may not depend on educational levels.

The effect of income on antibiotic consumption has contradictory results in literature: Masiero *et al*. (2010) [11] found a correlation between higher antibiotic use and higher GDP per capita across 17 European countries. However, poorer cantons presented higher antibiotic consumption in Switzerland [9]; and an analysis of prescription rates in Scotland demonstrated that patients living in the lowest SES quintile areas used 36.5% more antibiotics than those living in the highest SES quintile areas. [34] In the present study, GDP per capita or the level of income inequality (measured by the Gini coefficient) were not associated with consumption level in the multivariable regression model. However, HDI—an index associated with social and economic development—was the best predictor of higher antibiotic consumption in the univariate analysis. HDI, a composite measure of education, health and income, is thus probably associated with antibiotic consumption through its education and health components—life expectancy was, as education, found to predict higher consumption.

Percentage of population living in urban areas was the variable found to have the greatest impact on the multivariable model of antibiotic consumption. Ternhag *et al*. (2014) [35] reported that those living in urban areas in Sweden were prescribed more antibiotics than those living in rural areas. Possible reasons for a greater consumption in urban areas of SSP are higher self-medication rates due to lack of time to attend medical visits, and lack of access to medical care in rural settings. [35,36] Percentage living in urban areas, a more distal determinant, has had about half the impact on consumption in the multivariable model when compared to the univariate model—half of its impact is thus possibly mediated by other determinants studied, such as density of private health establishments and educational levels, which are more proximal and probably greater in urban areas. Population density and living in São Paulo city’s metropolitan area lost their effect after adjustment, and the percentage of population living in urban areas may have been responsible for their effect in the univariate models.

Higher density of private health establishments and lower density of SUS health establishments were associated with antibiotic consumption. These results are in the expected direction, considering that patients that attended the public sector already needed prescriptions before 2010 and that private doctors may be more eager to meet their patients’ demand for an antibiotic. [5] Density of SUS health establishments lost its effect in the multivariable model, and its association with consumption in the univariate model probably occurred due to confounding factors. Moura *et al*. (2015) [32] found no effect of the 2010 law on public channels in Brazil.

Demographic structure of the population has been widely analysed to date as a possible predictor of antibiotic consumption, with many different results being achieved. People under 5 and over 75 years old were prescribed more antibiotics in Sweden [35]; while the share of population over 65 years was negatively associated to antibiotic consumption in Switzerland [9]; and there was a positive association between consumption across Europe and population aged 14–25 years or 65–79 years. [11] Consumption was positively associated to population under 15 years old but negatively associated to population over 65 years old in British Columbia, Canada [10]; and no similar association was found in Hungary. [37] In our study, the percentage of population between 5 and 15 years old was negatively associated with consumption, but no association was found with percentage of population over 60 years old. The percentage of population under 5 years old was also negatively associated with consumption in the univariate model. Furthermore, a higher percentage of females in the population of SSP’s municipalities was associated with a higher antibiotic consumption—a finding that also agrees with previous work [35]. This may be related to the fact that females between 15 and 64 years are the group most commonly affected by urinary tract infection, a major cause of empiric oral antibiotic use and thus of AMR. [26] Not only antibiotic consumption was greater, but the policy was also found to be more effective in those municipalities with a higher percentage of females, possibly reducing the OTC use of antibiotic for this infection.

Even though the policy effect was greater in municipalities with previously higher consumption, it did not seem to be much related with the SED analysed—only the percentage of females in the population presented a good association in the multivariable model, with very little correlation with the data. This policy was previously described to be more effective in areas with better SES within Brazil [32]. It is not known whether the effect is not correlated with these SED mainly in SSP, which has overall good standards of living when compared to some parts of Latin America [28,38], or whether this is true for most areas affected by these policies. It would have been useful to have analysed the effect of physician density—it could help to predict future changes caused by improved access to physicians in areas previously lacking medical professionals. Furthermore, if there are areas in SSP with a shortage of access to physicians, the prohibition of OTC sales could incur in restricted access to antibiotics. Masiero *et al*. (2010), Filippini *et al*. (2006) and Marra *et al*. (2010) have all found that antibiotic consumption was higher in areas of higher physician density. [9–11]

Additionally, a more objective measure of incidence of infectious diseases would have been incidence of a specific disease such as *Campylobacter* infection, keeping consistency with previous studies [9]. The variable used in this study, number of hospital admittances due to infectious diseases, although measured by municipality of residence and not of treatment, may be influenced by differences in access to hospitals. We, however, had not access to this measure or to physician density.

European Union countries monitor their use of antibiotics through the European Surveillance of Antimicrobial Consumption project, which aims to produce international data on antibiotic consumption using information from sales and reimbursement data, encompassing all antimicrobials being consumed in the country (in community and hospital sectors) in order to guide possible public health policies. [6] LAC do not have a similar system, and thus wholesale pharmacy sales data are used to monitor antibiotic consumption. Although these data are able to produce rough estimates on level and trends of antibiotic use, they under-represent total antibiotic consumption, as the antibiotic dispensing happening in the public sector is not considered; [20,33] and may fail to detect possible trends caused by the new policy, such as apparently reducing antibiotic use but only shifting consumption from legal to illegal establishments, where consumption would not be measured. A surveillance system similar to the one available in European Union would be necessary to obtain more reliable data.

A limitation associated with this study is that its results cannot be extrapolated to the individual level. However, for the main purposes of this study and for the main implications for policy, the municipality level may be the most important. It is also important to remind that this study did not aim to investigate causation—it is not clear which of the associated factors, if any, were truly causative. Measurement error of the outcome may have occurred if pharmaceutical volume coverage differs across meso-regions/municipalities. Moreover, not all antibiotics are consumed in the municipality where the sale has occurred, and not all antibiotics are consumed within the month of its purchase. These factors may have increased misclassification of the municipality or month of consumption. Some variables may not be a good predictor at the municipality level—for instance, GDP per capita, as, at a much greater extent than at country level, not all income generated in a municipality goes to its own population. The addition of several variables in the multivariable models may have increased the amount of uncertainty and caused an over-adjustment of intermediate variables as some of them possibly mediated the effect of others. The use of other medication group as a control would have improved the analysis and dismissed possible secular trends. Finally, the large differences in municipality sizes may have caused the standard errors of this study to have been under-estimated.

## Conclusion

As in most other LAC where OTC sales have recently been restricted, the policy has changed the trend of increase and effectively reduced antibiotic consumption in SSP. Antibiotic consumption continued to decline in the second year after the policy was issued, and in 2012 it had lower levels than in 2008. SSP had similar levels of consumption to those yielded by literature for the whole country of Brazil. [15,20] SED of a higher oral antibiotic consumption in municipalities of SSP were: HDI, percentage of urban population, density of private health establishments, lower percentage of population between 5 and 15 years old, lower illiteracy levels, higher life expectancy and percentage of females in the population. The policy effect was stronger in municipalities with a higher percentage of females. More studies should be performed during the next years to analyse if the reduction in consumption is just an immediate effect of the policy or if it will endure.
